# Counselling preparedness and responsiveness of industrial psychologists in the face of COVID-19

**DOI:** 10.4102/sajip.v47i0.1860

**Published:** 2021-05-17

**Authors:** Marieta du Plessis, Emma C. Thomas

**Affiliations:** 1 Department of Industrial Psychology, Faculty of Economic and Management Sciences, University of the Western Cape, Cape Town, South Africa

**Keywords:** COVID-19, mental health, counselling, industrial psychology, preparedness, responsiveness

## Abstract

**Orientation:**

The coronavirus disease 2019 (COVID-19) pandemic has brought to the forefront the need for industrial-organisational psychologists (IOPs) and organisations to place an emphasis on employees’ mental and physical health at all times.

**Research purpose:**

The purpose of the research was to determine how prepared IOPs are to counsel employees during the pandemic and how responsive they are to provide counselling.

**Motivation for the study:**

It is not clear to what extent such counselling is being practised by IOPs in the workplace during the COVID-19 pandemic.

**Research approach/design and method:**

A qualitative approach was used to gain an understanding of registered South African IOPs’ experiences of workplace counselling, particularly during the time of the COVID-19 pandemic.

**Main findings:**

Regarding preparedness, we found that IOPs are ill-prepared to counsel in the workplace. Preparedness was influenced by participants’ counselling education, skills and knowledge; experience; convictions about counselling; and psychological and organisational preparedness. Whilst some IOPs did engage in more counselling during the COVID-19 pandemic, most reverted to mitigating actions such as referrals, wellness management, equipping managers and change initiatives.

**Practical/managerial implications:**

The results of this study indicate that, under pandemic conditions, there is an increased need for counselling practices within the workplace and that IOPs should explore the ways in which they could play a more active role in such counselling.

**Contribution/value-add:**

Although we found that IOPs generally responded to employees’ mental health needs in a positive manner, there was a lack of counselling preparedness and responsiveness during the COVID-19 pandemic.

## Introduction

The World Health Organization (WHO) declared the outbreak of coronavirus disease 2019 (COVID-19) as a pandemic in March 2020. For much of the following year, the COVID-19 pandemic had a bewildering and unprecedented effect on all aspects of society across the globe (Gautam & Sharma, 2020). For instance, in March 2020 the South African government implemented drastic measures to curb the spread of the virus, breaking existing social and economic forms of contact (Arndt et al., 2020). It quickly became evident that the direct and indirect psychological and social effects of the pandemic were pervasive and could affect mental health long after the pandemic itself is over (Holmes et al., 2020). As a result of prolonged lockdown and business closures, people experienced social isolation, lifestyle disruptions and loss of personal income, whilst society lost its productivity in a crippled economy (Tan et al., 2020). This pandemic has exacerbated stressors in a healthcare system in which burnout, a response to workplace stress, is already endemic (Restauri & Sheridan, 2020).

Given this global impact of the COVID-19 pandemic, there is much speculation about the effects it will have on the future of work and on people working in organisations (Rudolph et al., 2020). Indeed, the influence of the pandemic has provoked a career shock in many people (Akkermans, Richardson, & Kraimer, 2020; Hite & McDonald, 2020). There have been tangible effects on work-related processes, including job losses and large-scale implementation of new remote labour policies (Adalja, Toner, & Inglesby, 2020).

These changes have imposed numerous psychological stressors upon individuals (Van Bavel et al., 2020). For example, people are experiencing increased work and family demands, especially as they navigate the need to re-balance multiple work-related roles with their personal lives. Frontline employees such as healthcare workers needed increased levels of resilience, as they continued to attempt to save lives whilst battling snowballing numbers of infected people (Hite & McDonald, 2020). External demands (e.g. increased uncertainty about job security, financial difficulties) are likewise accumulating. Sibley et al. (2020) reported that the nationwide lockdown in New Zealand resulted in higher rates of mental distress. Zacher and Rudolph (2020) reported decreases in life satisfaction and positive affect in a German sample (*N* = 979). In India, a 20% increase in patients with mental illness has been reported since the COVID-19 outbreak (Loiwal, 2020).

This emerging evidence of the impact of the COVID-19 pandemic on mental health echoes WHO concerns about its long-term psychosocial consequences (WHO, 2020a). Specifically, there are concerns about increased experiences of loneliness, anxiety, depression, insomnia, harmful substance use, self-harm and suicidal behaviour (WHO, 2020b). Kumar and Nayar (2020) suggest that one of the major challenges in mitigating mental health consequences of the pandemic is the lack of mental health professionals, practitioners and counsellors.

Industrial-organisational psychologists (IOPs) are professionals who specialise in the psychology of work and human behaviour in organisations (Van Vuuren, 2010). The *Health Professions Act* (2006) (Health Professions Council of South Africa [HPCSA], 2011) postulates that the main tasks of IOPs are to:

[*P*]lan, develop and apply paradigms, theories, models, constructs and principles of psychology to issues related to the world of work in order to understand, modify and enhance individual, group and organisational behaviour, well-being and effectiveness. (p. 9)

Hence, IOPs should support well-balanced employees towards a process of development and optimisation. Although a key focus for IOPs is to ensure workplace readiness and compliance with occupational health and safety measures (Rudolph et al., 2020), it is clear that COVID-19 workplace interventions should address not only the physiological but also the psychological needs of employees (e.g. via counselling procedures) (Zhou et al., 2020). The purpose of this study was to determine the preparedness and crisis responsiveness of IOPs as related to workplace counselling.

## Research purpose and objectives

Traditional views of counselling suggest that this process should aim to help people overcome problems they are facing. When translated into the workplace sphere, counselling aims to help employees remain productive whilst coping with their problems. In the context of the COVID-19 pandemic, early counselling intervention could prevent the establishment of maladaptive cognitive or behavioural patterns amongst employees (Tan et al., 2020). Furthermore, counselling could help an individual increase their job resources, work engagement and psychological strengths (McLeod & Henderson, 2003).

It is not clear to what extent such counselling is practised by IOPs in the workplace during the COVID-19 pandemic. It is also not clear whether IOPs have sufficient or adequate training in and exposure to the particular set of skills (e.g. the ability to adapt to the needs of their clients, especially as those needs are affected by the external environment) that will allow them to enhance positive states through counselling.

Therefore, framed within the commonly used model of emergency management (Kapucu, 2008), the purpose of the research was to determine how *prepared* IOPs are to counsel employees during the pandemic (i.e. whether they have the capabilities necessary to react to a series of disruptive future events), and how *responsive* they are to the needs of their clients (i.e. whether they are able to react quickly and positively). Preparedness may be enhanced by education, practice, self-awareness and other opportunities offered through postgraduate studies, internship and work experience as an IOP. Responsiveness relates to well-being promotion, prevention and appropriate reaction to the legitimate expectations of employees, specifically during the COVID-19 pandemic.

In summary, this study had these specific objectives:

- To determine the preparedness of workplace IOPs to react to current mental health needs of employees, especially in the context of the COVID-19 pandemic.
- To explore the responsiveness of workplace IOPs as it relates to counselling during the COVID-19 pandemic.

## Literature review

A central component of workplace counselling is discussing with an employee those problems that have an emotional content. The aim is to help the individual cope within the situation; improvement in their coping skills often means improved efficiency of the organisation (Rothmann & Cilliers, 2007). Workplace counselling also often entails the application of brief, relationship-focussed psychological interventions. This application is consistent with HPCSA (2019)-mandated requirements for IOPs, which state that they should focus on ‘short-term counselling and helping skills, rather than longer-term interventions’ (p. 7). Barkhuizen, Jorgensen and Brink (2014) report that IOPs are well positioned to take on the role of facilitator in counselling situations, providing guidance, offering help and overseeing the intervention process. The focus is therefore to assist the employee in regaining control and responsibility over their life. Such interventions have been proved to be effective systemically and individually (McLeod, 2020).

Typical workplace counselling situations that IOPs face include career advising, developmental coaching, employee assistance (e.g. helping employees deal with ill-health diagnoses, workplace violence or substance abuse), guidance of progress towards resolution of personal, marital or relationship problems and facilitation of employees’ understanding of psychometric test results. Harassment, emotional abuse and victimisation in the workplace may also be addressed (Aquino & Thau, 2009). Form 218-INDS (HPCSA, 2019) further indicates that IOPs should be exposed to the diagnosis of work-related stress, burnout, depression and psychological trauma (see also Preece, Cayley, Scheucl, & Lam, 2005).

The literature suggests, however, that even before the COVID-19 pandemic, IOPs did not feel adequately prepared for their role as workplace counsellors (Barnard & Fourie, 2007; Jorgensen-Graupner & Van Zyl, 2019). This feeling could be ascribed, at least partly, to the lack of structured guidelines, models, frameworks or meta-theories to inform workplace counselling (Barkhuizen et al., 2014). Alternatively, it seems that many IOPs feel uncomfortable with their level of competence in counselling and may prefer not to counsel employees, because of the perception that such a role falls outside their scope of practice (Jorgensen-Graupner & Van Zyl, 2019). However, one of the contemporary roles of the IOP is that of wellness facilitator (Van Zyl, Nel, Stander, & Rothmann, 2016). This role includes enhancing the overall level of personal well-being of employees, to prevent workplace problems (Barkhuizen, Jorgensen, & Brink, 2015). It seems natural, therefore, that part of this role could include the provision of counselling to address the personal and/or professional developmental needs of the individual employee (Van Zyl et al., 2016).

As a psychologist in the workplace, the IOP often serves as the ‘first responder’ to help employees manage job-related distress and trauma (Barkhuizen et al., 2015). The responsiveness of the IOP in these cases may range from engaging directly with the employee to provide crisis counselling, to facilitating interventions on individual, environmental, organisational and group levels (Georganta & Montgomery, 2019; Jonker, Graupner, & Rossouw, 2020). Terblanche and Van Wyk (2014) posited that organisations should implement effective psychological trauma management programmes and interventions, to ensure that employees are able to maintain their levels of work performance and well-being. Large-scale disasters, such as the COVID-19 pandemic, are associated with significant increases in mental health disorders (e.g. post-traumatic stress disorder [PTSD], depression and substance abuse disorders) in both the immediate aftermath of the trauma and subsequently (Galea, Merchant, & Lurie, 2020). Similarly, burnout is associated with higher rates of substance abuse, depression and suicide (Dyrbye et al., 2014). Hence, it is clear that the IOP’s responsiveness in situations of trauma and burnout is paramount to employee and organisational well-being. Industrial-organisational psychologists can help employees manage stress before it results in burnout, trauma debriefing can lessen the chances of anxiety or mood disorders developing and building on the employee’s personal resources can help buffer potentially excessive job-related demands (Jorgensen-Graupner & Van Zyl, 2019).

### Research design and method

We used a qualitative, interpretive phenomenological approach to achieve our aim of gaining an in-depth understanding of registered South African IOPs’ experiences of workplace counselling, particularly during the time of the COVID-19 pandemic. This approach helps the researcher understand and interpret the meanings that participants attach to everyday life as they experience it, and as such it was appropriate to the aims of the study (De Vos, Strydom, Fouche, & Delport, 2005; Gray, 2004).

We applied the phenomenological method by engaging with the participants in a naturalistic manner (i.e. by having a discussion with each individually). By using this strategy, we could explore the experiences of a number of participants working in different organisations. As a result of social distancing measures associated with the COVID-19 lockdown regulations, face-to-face interviews and focus groups were discouraged. Thus, online individual interviews were the best option to learn about the participants’ experiences.

#### Research strategy

Participants were HPCSA-registered IOPs from across South Africa and recruited by using convenience and snowball sampling. A criterion for inclusion was that the participant had to be registered with the HPCSA in the category ‘Psychologist: Industrial’.

#### Research setting

Participants were interviewed via video conference, with the interviewer and interviewee at their homes or offices. The interviews were conducted in August 2020, a month during which South Africa was in alert level 3 of the COVID-19 lockdown. This alert level mandated restrictions on many activities, including social and work-related activities, for example, persons who are able to work from home should do so (Department of Co-operative Governance and Traditional Affairs, 2020).

#### Entreé and establishing researcher roles

Individuals who conducted the study were two principal researchers (i.e. they conceptualised the study, analysed the transcripts and wrote the research report) and 22 master’s students who acted as fieldworkers (i.e. they conducted the interviews). The students were registered for and actively pursuing their master’s degree in industrial psychology.

The principal researchers are both registered IOPs and are involved in teaching a master’s-level module on workplace counselling. In considering the impact of those roles on study outcomes, we acknowledge that: (1) our experiences as practising IOPs may influence data interpretation and (2) we may carry a positive bias towards counselling preparedness and responsiveness of IOPs in the workplace. Another important consideration in this regard is whether participants were aware of the fact that fieldworkers were aspiring IOPs as this might have influenced their answers (and especially their willingness to openly talk about their experiences).

Researchers and fieldworkers gained access to participants by contacting individuals in their professional networks and by searching for registered IOPs on various Internet platforms (e.g. LinkedIn, Google). Participation invitations were sent via e-mail or online messages. Individuals who responded positively were asked to indicate a willingness to consent to take part in an interview. For those who did so, the interviewer arranged a date, time and suitable virtual platform for the online interview.

Prior to beginning the interview, participants were informed about the amount of time and level of participation required. A formal consent letter described the nature of the information that would be gathered, its intended use and role players who would have access to the data. It also assured participants that they could withdraw from the study at any time without fearing repercussions. No form of coercion or dishonesty was used to ensure participation or to elicit responses.

#### Participants

Table 1 presents basic demographic characteristics for the 23 participants (17 women and six men). Eight were self-employed and 15 were employed full-time in industries including financial services, public sector, fast-moving consumer goods (FMCGs), medical services and human resource (HR)- and IOP-related consulting firms. All participants were employed in IOP- or HR-related work, with job titles such as HR Business Partner, Chief HR Officer, Organisational Development (OD) and People Development, HR Advisor, HR Manager and Assessment Manager. Participants had been registered as an IOP for between 3 months and 36 years (average = 10 years). However, many participants had substantial work experience in an HR role before they furthered their studies and registered as an IOP.

**TABLE 1:** Participants’ demographics.

Participant code	Gender	Years as an IOP	Employment status
P1	Female	3.5	Permanent employment
P2	Female	3	Permanent employment
P3	Male	32	Permanent employment
P4	Female	6	Permanent employment
P5	Female	3.5	Self-employed
P6	Female	9	Self-employed
P7	Male	18	Self-employed
P8	Female	7	Permanent employment
P9	Female	17	Self-employed
P10	Female	2	Permanent employment
P11	Female	5	Permanent employment
P12	Female	0.3	Self-employed
P13	Female	7	Permanent employment
P14	Male	34	Permanent employment
P15	Female	5	Self-employed
P16	Female	15	Permanent employment
P17	Male	1	Self-employed
P18	Female	14	Self-employed
P19	Female	3	Permanent employment
P20	Male	1	Permanent employment
P21	Male	36	Permanent employment
P22	Female	1	Permanent employment
P23	Female	5	Permanent employment

IOP, industrial-organisational psychologist.

### Data collection methods

We used semi-structured one-on-one interviews to collect data. The purpose of the interviews was to elicit thorough information relating to the experience and perception of participants about their preparedness for workplace counselling and counselling responsiveness during the COVID-19 pandemic. The interview guide contained questions such as:

‘How did the expectations of your work as an IOP change as a result of the COVID-19 pandemic?’

‘Have you been doing more counselling during the pandemic than before?’

‘If a second wave of the COVID-19 pandemic hits, what would you like to have in place that you did not before?’

The interview guide also contained demographic questions and asked for information about the participant’s type of employment, size of the employing organisation and years of registration as an IOP.

### Data recording

Interviews were recorded digitally by using the recording function of the online meeting platform. The interviews were then saved in electronic format, with back-up copies in a cloud-based drive. The data were transcribed in Microsoft Word documents and then compared with the voice recordings to ensure no omissions.

### Data analysis

We used the interpretive phenomenological analysis steps described by Smith and Osborn (2008) to analyse the data. As such, all transcripts were subjected to a systematic search for themes. Codes were generated from the data to annotate insights into participants’ experiences. Emerging patterns across cases were sought and classified as themes. Themes were then grouped under superordinate (broad) themes.

### Reporting style

We used a narrative reporting style to describe and explain the identified themes. The themes are described in detail, supported with extracts from the transcripts (Smith & Osborn, 2008). To assist the reader, minor modifications were made to transcripts, as long as it did not impact on the meaning of what was said; for example, we omitted interjections and duplicated words from the transcripts (De Vos et al., 2005).

### Ethical considerations

Participant identity was anonymised throughout, as names were not used during data collection. No private information was elicited, and the right of confidentiality was respected for all participants. Ethical clearance was obtained from the Human and Social Sciences Research Ethics Committee of the University of the Western Cape, with clearance number HS20/7/1.

## Findings

Participants were asked to reflect on how they experienced their beliefs, feelings and responses with regard to workplace counselling during the COVID-19 pandemic. Their accounts were organised in two superordinate themes to reflect preparedness (i.e. experiences, perceptions and knowledge evident at the onset of the COVID-19 pandemic) and responsiveness during the pandemic. To contextualise the findings, we begin with a report of how IOPs experienced the need of employees and organisations during the COVID-19 pandemic.

### Employee and organisational needs experienced during the COVID-19 pandemic

Participants’ accounts of the workplace experience during the COVID-19 pandemic indicate that employees experienced uncertainty and anxiety (P3, P6, P22). Some of these were as a result of job insecurity (P3), partners and/or family members losing their jobs or falling ill (P8), perceptions of the deadliness of the virus (P3) and lack of certainty in a time when none existed (P8). The trauma of losing a family member also had a dire impact on some employees’ well-being (P10), especially if the loved one passed away in isolation and there was no opportunity to say goodbye (P13). Participant 6 indicated that the needs of employees changed with the different levels of lockdown:

‘If level 5 represented creating a sense of certainty with information, level 4 was about safety, level 3 was about social awareness. Level 2 now is about how you are going to get people to regulate; it is not yet a normal reality.’ (P6, 9 years as IOP, self-employed)

The need to work from home was also challenging for many employees, indicating that many felt overwhelmed by the impact on their family life. Participant 1 relates that employees asked:

‘Is this the way I’m going to work for the rest of my life and my reality.’ (P1, 3.5 years as IOP, permanently employed)

‘You have to cook whilst you’re at home, you have to do your office work, and you have to take care of your family and, and, and ….’ (P19, 3 years as IOP, permanently employed)

Participants related that the home–work environment was further challenged by the need to provide home schooling for children and the experience of longer working hours:

‘[*A*] working day that is longer because of the fact that they are online the whole day.’ (P1, 3.5 years as IOP, permanently employed)

‘People’s routines and boundaries are completely interrupted and things feel very much out of control.’ (P23, 5 years as IOP, permanently employed)

Some of this was related to technology, stating:

‘[*T*]echnology is fantastic, but if people don’t respect your time it creates stress.’ (P1, 3.5 years as IOP, permanently employed)

The home–work relationship issues also became pertinent, as:

‘[*P*]eople confronted and having to live in close proximity have to face their issues.’ (P20, 1 year as IOP, permanently employed)

Furthermore, pragmatic difficulties were experienced, for example, that you:

‘[*C*]an’t go isolate in a shack of six people.’ (P16, 15 years as IOP, permanently employed)

Frontline staff, who needed to continue in their roles as essential service workers, feared going home, given the risk of infecting their families with the virus (P3).

The resultant psychological effect on employees became evident. Employees experienced social isolation. Participant 1 indicated that there was an increase in calls to the employee assistance programme, but:

‘[*P*]eople still want the face-to-face interaction or even a colleague or a friend.’ (P1, 3.5 years as IOP, permanently employed)

‘I think the second pandemic, after COVID, will be mental health … people are sometimes just not coping.’ (P13, 7 years as IOP, permanently employed)

For some, the changes were experienced more severely. Other participants related:

‘I’ve had to deal with four cases of people who are on the brink of committing suicide.’ (P3, 32 years as IOP, permanently employed)

‘[*T*]here is definitely a spike in depression.’ (P15, 5 years as IOP, permanently employed)

This was echoed by another who indicated:

‘[*T*]he type of things we are seeing are PTSD, high increase in anxiety disorder, also within the virtual work environment, major depressive disorder….’ (P19, 3 years as IOP, permanently employed)

The results of the rapidly changing environment also include burnout (P18, P19).

For organisations there was a need to adjust rapidly. A major change included the application of virtual work environments through the use of technology (P21). Participant 4 related:

‘We had to make sure that we were properly resourced from a computer perspective, internet connection, we had to update our manuals, our processes and our documents.’ (P4, 6 years as IOP, permanently employed)

With employees working from home, there was also the:

‘[*C*]hallenge to look at policies and our procedures and to say how are we going to reward people, you know, we need to make sure that people still follow our protocols whilst at home.’ (P3, 32 years as IOP, permanently employed)

Implementation of social-distancing measures and hygiene practices also needed to be incorporated (P3).

### Preparedness

Referring to the many crises caused by the COVID-19 pandemic, Participant 1 succinctly stated: ‘I don’t think any training could have prepared us for the situation we are currently in’. Most other participants agreed that they felt ill-prepared to deliver mental health counselling services required by employees. Within the major theme of preparedness we identified the following subordinate themes: education, skills and knowledge; experience; convictions about counselling; psychological preparedness; and organisational system preparedness.

#### Education, skills and knowledge

To summarise, the general sentiment as:

‘I would say I didn’t get enough training on counselling.’ (P23, 5 years as IOP, permanently employed)

It seems that the problem is pervasive in the South African context because:

‘IOP training at university level is not standardised, and that is a challenge because some universities include counselling in their master’s programme and others don’t.’ (P19, 3 years as IOP, permanently employed)

Participant 5 confirmed this in their training by stating:

‘[*T*]here was no counselling course.’ (P5, 3.5 years as IOP, self-employed)

Participant 12 also indicated that the focus of IOP training may be wrong:

‘We focus so much on the business side of things in psychometrics that we actually didn’t do much on counselling.’ (P12, less than a year as IOP, self-employed)

Some participants who did have counselling as part of their training felt that more *practical exposure* (P4) and *practice counselling* (P5) were required. Another expressed the view that:

‘[*W*]e are very behind; we are still learning the same theories from the last 30 years.’ (P1, 3.5 years as IOP, permanently employed)

One participant felt that theory does not help one to prepare, but:

‘I see preparation as taking on a counselling role, and less of a focus on counselling theories. Preparing you to think like a counsellor.’ (P10, 2 years as IOP, permanently employed)

Some participants mentioned that they gained additional training to complement their skills in counselling:

‘I have a clinical supervisor who has in our feedback session shown me how to counsel.’ (P18, 14 years as IOP, self-employed)

Other participants expressed the need for a reform of IOP training at the master’s level to include:

‘[*D*]ealing with your first level of trauma in the workplace.’ (P19, 3 years as IOP, permanently employed)

Others expressed the need for psychopathology (P7), containment (P5) and identifying emotional problems (P3).

#### Experience

Participant 12 shared:

‘I haven’t had to do any counselling yet. And I think I actually don’t feel like I’m prepared for it.’ (P12, less than 1 year as IOP, self-employed)

Although one reason for lack of experience and exposure to counselling may be tenure as an IOP, another reason seems to relate to the roles that registered IOPs fulfil in organisations. Participant 22 said:

‘So I think most industrial psychologists work as HR managers or as some form of HR personnel within companies.’ (P22, 1 year as IOP, permanently employed)

Participant 13, referring to counselling experience, said:

‘That’s really not my role. It’s more of corridor chat, you know?’ (P13, 7 years as IOP, permanently employed)

Participants also observed their lack of counselling experience in the online space (P20, P21). Participant 5 related:

‘There were a lot of issues. We had to look into confidentiality; others shouldn’t be able to see.’ (P5, 3.5 years as IOP, self-employed)

Participant 20 supported the importance of confidentiality whilst simultaneously noting the difficulties inherent in online counselling:

‘It’s important to create a safe space for somebody and almost like create a hub of confidentiality. Online, it changes because you don’t see the body language as much.’ (P20, 1 year as IOP, permanently employed)

#### Convictions about counselling

Here, conviction is understood as the subjective stance about your beliefs, and confidence that this belief is accurate. We identified this theme because many participants had a strong opinion about their responsibility/legitimacy to counsel. Participant 17 noted that:

‘[*Y*]ou always have to take into mind the category of registration and also the limits of the category of registration … you are an IOP, so you do not give therapy, we are not trained to give therapy.’ (P17, 1 year as IOP, self-employed)

Even though this participant correctly states that therapy is not part of the scope of practice of an IOP (HPCSA, 2019), participants seem to categorise all forms of counselling as therapy. In some cases, individuals felt restricted by this perceived limitation on the scope of practice of IOPs:

‘I cannot work in any kind of counselling capacity, even though I have counselling training and that is something that drives me insane.’ (P18, 14 years as IOP, self-employed)

At the other end of the spectrum, Participant 21 indicated:

‘In the training and curriculum of industrial psychology there’s too much emphasis on counselling and psychopathology. That is not the work of an industrial psychologist. It is the work of a counselling psychologist.’ (P21, 36 years as IOP, permanently employed)

Understandably, participants’ views about the responsibility/legitimacy of counselling have an impact on their engagement in counselling. Phrases such as *we cannot counsel* (P12, P16 and P18) and *we will not do the counselling* (P14) were pervasive across a number of interviews.

#### Psychological preparedness

Participants indicated that dealing with the employee and personal experience of the changes during the pandemic was difficult. Participant 5 stated:

‘I think there’s probably an expectation that you need to be able to respond to that [*refers to issues weighing on him or her*] and cope with that, which we’re not always prepared for.’ (P5, 3.5 years as IOP, self-employed)

There was also much pressure and:

‘[*T*]here was no room to relax, so everyone had to really push.’ (P8, 7 years as IOP, permanently employed)

Participant 3 related:

‘Being an industrial psychologist you think you are strong enough to deal with these issues personally.’ (P3, 32 years as IOP, permanently employed)

With some dire effects:

‘I have had to take some medications to calm me down just to keep focus … [*and*] … have been having sleepless nights for the last 3 to 4 months.’ (P3, 32 years as IOP, permanently employed)

‘I don’t think I’ve cried as much as I did in the last few months … I was challenged to look at my own mental health in the sense of I wasn’t sure about my own job. I still needed to take care and look out for other people’s jobs.’ (P22, 1 year as IOP, permanently employed)

Participant 10 summarised:

‘You just have to react and the business comes first. So, I wish we were trained to be able to adapt.’ (P10, 2 years as IOP, permanently employed)

However, other participants indicated how they were able to deal with the situation:

‘I have adapted and ran with things … I had to by myself, pick myself up.’ (P15, 5 years as IOP, self-employed)

‘IOPs know more about EQ [*emotional int elligence*] to be able to take a step back and manage ourselves.’ (P22, 1 year as IOP, permanently employed)

Having a counsellor, coach or mentor to:

‘[*H*]old you accountable for your own emotions and your own struggles.’ (P22, 1 year as IOP, permanently employed)

#### Organisational system preparedness

Participant 2 expressed a wish:

‘If we should ever go through a second pandemic again, I would rather prefer as an industrial psychologist that the workplace be better equipped for the situation.’ (P2, 3 years as IOP, permanently employed)

This statement provides evidence for IOPs’ need for adequate support and resources to operate effectively in the organisation (Barnard & Fourie, 2009). Specific matters regarding social distancing had an influence, as Participant 20 observed:

‘[*M*]ore than two people are not allowed in our office, so counselling is difficult.’ (P20, 1 year as IOP, permanently employed)

On the other hand, some participants expressed frustration at being under-resourced to assist:

‘[*T*]wo industrial psychologists with almost 6000 employees. It’s impossible.’ (P3, 32 years as IOP, permanently employed)

Policies, practices and procedures to effectively manage and support a work-from-home model were mentioned by participants 2, 7, 12 and 20. Noticeably, no participant made reference to the creation or adaptation of trauma and crisis management policies.

### Responsiveness

The questions posed to participants were framed specifically around their counselling responsiveness in the workplace in the face of COVID-19. Their answers to these questions made it evident that these IOPs used other approaches, in addition to or in place of counselling, to improve employee well-being in the workplace. Therefore, within the major theme of responsiveness, we identified the following subordinate themes that reflected these mitigating approaches: referrals, general wellness initiatives, equipping managers, corporate social responsibility and change management. Before elaborating on participant responses that supported the identification of those subordinate themes, we consider responses that referred specifically to counselling activities.

#### Counselling

The counselling responses tended to be addressed at experiences of PTSD (P17), career advising (P18), work–life integration (P9), dealing with home circumstances (P17) and managing change and anxiety (P5). Some participants indicated that they engaged in more counselling during the pandemic (P5, P9, P17, P18 and P19). Specifically, when asked: ‘Do you have more counselling sessions now’, two participants answered that:

‘Yes, I am doing quite a few coaching sessions across the organisation.’ (P8, 7 years as IOP, permanently employed)

‘I have done that kind of coaching and discussions and sessions and stuff like that.’ (P11, 5 years as IOP, permanently employed)

Note here that these participants overtly classified counselling as being synonymous with coaching. Other participants preferred using the term check-in, which was explained as:

‘[*E*]lements of counselling which we call catch-ups or check-ins.’ (P10, 2 years as IOP, permanently employed)

That these participants preferred to talk about ‘coaching’ or ‘check-in’ sessions, rather than counselling, is likely consistent with a theme identified earlier (i.e. that these IOPs felt ill-prepared to engage in what they regarded as traditional long-form counselling or even: ‘A short-term intervention-focussed counselling programme’ [P19]).

Similarly consistent were statements explaining that workplace interventions were:

‘[*N*]ot full counselling sessions, but taking care and chatting to and establishing better programmes for them.’ (P21, 36 years as IOP, permanently employed)

Although IOPs do not engage in counselling:

‘[*W*]hen you see improvement, you see people regaining strength, becoming self-motivated – I’ve realised that if people talk, it improves and greatly influences the healing process.’ (P2, 3 years as IOP, permanently employed)

#### Referrals

Numerous participants mentioned that the major outlet to whom they referred individuals for counselling were the employee assistance programme (EAP) (P1, P2, P3, P10, P13 and P16). Participant 3 gave one instance of such a referral:

‘The mother tested positive, the mother is one of our employees. When she communicated that to her daughter at home, the daughter tried to commit suicide. Fortunately, we made use of an employee assistance company.’ (P3, 32 years as IOP, permanently employed)

Participant 10 reported having conversations to:

‘[*C*]onfirm what some of the issues are that are impacting performance, and then referring people to our employee assistance programme.’ (P10, 2 years as IOP, permanently employed)

In many cases, the scope of the EAP also included services to family members of employees, as Participant 19 explained:

‘[*Y*]ou can refer someone for counselling; and or the family members, depending on who is entitled to use the services.’ (P19, 3 years as IOP, permanently employed)

In most cases, the EAP service was outsourced. Participant 19 explicitly stated:

‘[*W*]e have outsourced the counselling function. In our team we do the consulting and the management side of wellness.’ (P19, 3 years as IOP, permanently employed)

Some participants stated that, because an EAP was not available to them, they referred in-need employees to a network of mental health associates that included clinical and counselling psychologists (P14, P15 and P20).

#### Wellness management

Participant responses indicated that they took responsibility for employee wellness: ‘I’m the only Industrial Psychologist in the company, so I’m basically what we call in our world the “custodian of the EAP programme”’ (P10).

Whilst counselling was referred to EAP services, IOPs may have taken a more managerial role as it relates to wellness. Participant 10 indicated:

‘[I am] doing a mental health survey with people. It is more direct, and we can focus on the whole staff complement, as opposed to me or the line manager identifying someone potentially needing help.’ (P10, 2 years as IOP, permanently employed)

Other initiatives took the form of online workshops on topics such as stress management, resilience (P19), anxiety (P23), awareness of mental health (P10), taking care of those suffering from burnout (P19) and fitness sessions (P23). Participant 16, however, indicated that these approaches were not enough, stating that:

‘[*W*]e were too slow to identify what interventions were needed to support the organisation and the employees.’ (P16, 15 years as IOP, permanently employed)

#### Equipping managers

A number of participants needed to equip and capacitate managers to help them deal with difficult situations that arose during the COVID-19 pandemic. Participant 6 explained:

‘Line managers have become HR managers. They had to check in on the employee. So a lot of upskilling happened … now line managers have better behavioural knowledge and skills because the system forced it.’ (P6, 9 years as IOP, self-employed)

Clearly, the need to empower managers with these new skills was evident:

‘Many, many managers are very scared to manage employees with mental health conditions.’ (P19, 3 years as IOP, permanently employed)

Participant 19 further elaborated:

‘Let’s say a colleague passes away because of COVID, or someone has tested positive, and they were at work and everyone else was in the offices. Obviously, those people are traumatised – how would the manager deal with that?’ (P19, 3 years as IOP, permanently employed)

#### Corporate social responsibility

Apart from providing counselling and wellness assistance to employees, some participants mentioned looking after the needs of employees in a more communal manner:

‘Asking [*employees who are COVID-19 positive*] “are you guys coping?,” “do you have food?” things like that, because you might be living alone and can’t go out to get food.’ (P13, 7 years as IOP, permanently employed)

Participant 11 mentioned a similar scenario for employees who usually receive food at work, where the organisation:

‘[*C*]reated a little basket of food they could take home for the pandemic or something like that.’ (P11, 5 years as IOP, permanently employed)

Participant 8 mentioned:

‘[*W*]e do help others … letting people volunteer trying to reach out … to build this feeling that we were going to try and combat and support each other, not only internal, but external as well.’ (P8, 7 years as IOP, permanently employed)

Thus, whilst IOPs usually confine themselves to matters relating to the workplace, there was a realisation that:

‘[*Y*]ou cannot only deal with the person within the circumstance of work, the home circumstance has to be incorporated as well.’ (P17, 1 year as IOP, self-employed)

#### Re-evaluation and change

Participants mentioned ways in which the workplace circumstances forced on them by the COVID-19 pandemic compelled them to re-evaluate their roles and to make changes in their approaches to certain situations. Broadly speaking, the participants felt that:

‘The effects of COVID-19 have changed our perception of work … do we really need all the people at work all the time, and the answer is no.’ (P1, 3.5 years as IOP, permanently employed)

‘Moving from face-to-face learning facilitation to online learning was the biggest difference prior to the pandemic and compared with now.’ (P23, 5 years as IOP, permanently employed)

‘As an IOP, my work has changed in the sense of preparing the workforce for working remotely fully and at the same time ensuring business continuity.’ (P8, 7 years as IOP, permanently employed)

This includes considerations on whether the workforce has the necessary skills and personality to be able to work in a virtual environment (P1). In anticipation of this changed context, Participants 4 and 5 made similar statements when discussing organisational requests for assessments:

‘[*There is a*] great need from the business around assessing candidate’s competence or potential to be able to work remotely.’ (P4, 6 years as IOP, permanently employed)

‘What do we look for in people to know that they’ll be able to work from home unsupervised?’ (P5, 3.5 years as IOP, self-employed)

‘[*Remote working*] will have an influence on the [*desired*] skillset and personality.’ (P1, 3.5 years as IOP, permanently employed)

Because the pandemic’s effects on the workplace made employees reconsider the meaning and purpose of their career, several participants (e.g. P13 and P15) reiterated the need for investment in deep soft-skill building through coaching and transformational learning. Participant 8 expressed:

‘[*T*]here is a lot more career coaching than I anticipated, but I think it is people starting to think about, like, what do I want from life and where am I going.’(P8, 7 years as IOP, permanently employed)

## Discussion

This study set out to investigate how prepared IOPs are to react to the mental health needs of employees in the context of the COVID-19 pandemic, and how responsive they are to those counselling needs during this pandemic. Although our sample of 23 registered IOPs had received training at different institutions and had varying levels of professional experience, their responses during the semi-structured data collection interview were quite consistent. We reflect on the counselling responsiveness of IOPs during the COVID-19 pandemic. To explain the lack of counselling responsiveness of IOPs we present a discussion of the elements of preparedness evident from the thematic analysis. We conclude by acknowledging some of the study’s limitations and by giving recommendations for future research.

### Responsiveness of workplace industrial-organisational psychologists to employees’ counselling needs during the pandemic

Although a few participants did indicate that they engaged in more counselling sessions than before the COVID-19 pandemic, in most cases, IOPs did not opt to engage in counselling as a first response to employees’ mental health needs. Some participants also used the terms counselling, coaching and check-ins in a synonymous manner. Barkhuizen et al. (2014) correspondingly reported participants’ view that coaching is closely related to counselling. From an international perspective, IOPs are viewed as particularly qualified to provide coaching, and that many IOPs formally engage in coaching activities (American Psychology Association, 2013; Society for Industrial and Organizational Psychology, 2013). However, Koortzen and Oosthuizen (2010) caution that coaching and counselling require different approaches. For instance, whilst both approaches require active listening, coaching is more issue-focussed, takes various sources of information into consideration, and measures effectiveness of the coaching process through objective measures (in comparison with self-reported improvement through counselling). Hence, it is advised that counselling and coaching should be contracted separately with clients/employees (Interest Group in Coaching and Consulting Psychology [IGCCP], 2016) to avoid entering into potentially conflicting professional roles (Ethical Rule of Conduct, HPCSA, Form 223).

Participants who did not respond to employees’ well-being needs through counselling seemed to default to relatively familiar and well-practised IOP responses, such as referrals, equipping managers and change management processes. We classified these responses as mitigating actions. Framed within emergency management theory, mitigating actions help to prevent or lessen the impact of the disaster, whilst responsiveness would be to address the direct needs of employees (Waugh & Streib, 2006). The reported mitigating responses are all within the scope of practice of an IOP (HPCSA, 2019) and may have been effective in helping to mitigate the impact of the COVID-19 pandemic on the workforce. However, such responses did not directly address the mental health needs of employees through counselling. Therefore, we propose that a lack of preparedness is limiting the capability of IOPs to respond effectively to employees’ mental health counselling needs.

### How prepared are workplace industrial-organisational psychologists to react to employees’ mental health needs?

Our finding regarding preparedness is consistent with that reported by Barkhuizen et al. (2015), who stated that IOPs are ill-prepared to counsel in the workplace. This lack of preparation appears to have been brought into sharp relief by the increased need for mental health counselling by employees during the COVID-19 pandemic. Because the pandemic and associated government-mandated lockdown regulations have brought about tremendous change and uncertainty, people have had to adapt to and accept new norms quickly. Consequently, many people have experienced deteriorating mental health (United Nations, 2020; Vindegaard & Benros, 2020). Many participant responses indicated that, in their experience, employee anxiety and burnout have been heightened to the point that some workers have even shown signs of PTSD.

In the face of this increased need for mental health counselling in the workplace, responses from our sample of IOPs allowed identification of five distinct subordinate themes within the major theme of ill-preparedness.

The first related to education, skills and knowledge – participants indicated they had experienced a lack of adequate preparation for workplace counselling during their postgraduate training. This lack of training seems to run contrary to what is suggested in the literature. For instance, Pienaar and Roodt (2001) suggest that IOPs should emerge from their academic training fully equipped with counselling competencies (e.g. expressive and empathic communication and listening skills) that will help them operate ethically and effectively in the workplace (see also Barkhuizen et al., 2014; Ivey, Ivey, & Zalaquett, 2013; Johnson & Kaslow, 2014; McLeod, 2020)

The second preparedness subordinate theme was related to *experience* – participants indicated that, because they did not typically deliver mental health counselling services, they had little experience in doing so when called upon during the COVID-19 pandemic. It appeared that, under usual circumstances and even during the pandemic when there was a clear increase in the need for counselling, the IOP response was to outsource this function to clinical psychologists and other professionals. Hence, even those who had received adequate postgraduate counselling training may have experienced a lack of self-efficacy in this sphere because of a lack of experience (Barkhuizen et al., 2015). As a result of this lack of engagement in counselling practice by IOPs, one begins to question the extent to which the functions of the profession are being properly utilised within the workplace.

The third preparedness subordinate theme was related to *convictions about counselling* – participants indicated that mental health counselling did not fall within their scope of practice. This belief about the parameters defining their work-related activities is, broadly speaking, consistent with HPCSA guidelines stating that the scope of IOPs’ counselling interventions is limited to the ‘diagnosis of workplace psychopathology and the recognition of the need for further treatment and psychological intervention’ (HPCSA, 2019, p. 7). This limited form of (typically short-term) intervention, which can include post-trauma and crisis counselling, stands alongside other acts that fall within the scope of IOP practice: for example, planning, developing and applying paradigms, theories, models, constructs and principles of psychology in the workplace context to understand, modify and enhance organisational effectiveness (Barkhuizen et al., 2014). In other words, IOPs may provide basic counselling and act in a helping capacity when work-related dysfunction affects job performance. They may not, however, provide individual psychotherapy that extends outside that realm. When it does, they must refer the employee to another professional (e.g. a clinical psychologist) who can implement specific, possibly longer-term, interventions (HPCSA, 2019).

The fourth preparedness subordinate theme was related to *psychological preparedness* – participants indicated that, because they were struggling to cope with how the COVID-19 pandemic is affecting them, they were often not cognitively or emotionally capable of addressing the needs of employees who sought counselling services from them. When counsellors or therapists are experiencing compromised levels of personal emotional energies, they may be more vulnerable than ordinary individuals to psychological distress because of inherent qualities of compassion, empathy and caring (Lawson & Myers, 2011; Lawson & Venart, 2005; Skovholt, 2001). Moreover, because counselling sessions during the pandemic are more likely than those during ordinary times to be focused on traumatic events (e.g. the death of a loved one) that perhaps have been experienced by the IOP themselves, there is an element of shared trauma to the relationship (Bell & Robinson, 2013; Day, Lawson, & Burge, 2017). There is also, perhaps, a greater likelihood that IOPs who provide counselling services during the pandemic will be affected by at least some degree of vicarious trauma; that is, will not only bear witness to the employee’s experiences of a seriously distressing or tragic event, but will also take on the responsibility of intervening in that situation (Lerias & Byrne, 2003; Trippany, Kress, & Wilcoxon, 2004).

The fifth and final preparedness subordinate theme was related to *organisational system preparedness* – participants indicated that the companies that employed them, as well as the managers of the employees whom they were counselling, had put hastily constructed and often inadequate measures in place to deal with the pandemic. Studies describing institutional COVID-19 reactions indicate that organisations quickly had to revisit and change existing strategies, and had to be innovative, collaborative and resilient to prepare, respond and cope with the changing situations presented by this global pandemic (Barkai, 2020). For instance, many organisations shifted rapidly to remote modes of working, meaning that, almost overnight, their employees had to learn strategies that would enable them to work from home efficiently (Rudnicka et al., 2020). One of the challenges that came with this shift was establishing and maintaining a successful work–life balance. Industrial-organisational psychologists then played an important role in helping employees to establish such a balance and to integrate the two successfully. What also became apparent was organisational ill-preparedness with regard to trauma management policy and practice. In this regard, effective pro-active psychological trauma management programmes and interventions should be put in place (Jonker et al., 2020) by IOPs.

### Practical implications

The COVID-19 pandemic has shifted the definition of a workplace to incorporate, amongst others, agility, digitisation and workspace flexibility (Kaushik & Guleria, 2020). Industrial-organisational psychologists have to be cognisant of the way that these shifts, and the pandemic more generally, have affected employees’ emotional, behavioural and mental well-being. The results of this study indicate that, under pandemic conditions, there is an increased need for counselling practices within the workplace, and that IOPs should explore the ways in which they could play a more active role in such counselling.

Tertiary institutions, as well as other training facilities and organisations, can play an important role in preparing IOPs for such counselling activities. A first step for them might be an overall evaluation of the skills that might be lacking within the profession. Subsequent steps might include incorporation of additional practical and theoretical training to ensure that IOPs are equipped to handle a broader range of counselling situations.

Industrial-organisational psychologists themselves must, whilst remaining completely aware of and familiar with the limits imposed on their activities by the HPCSA’s scope of practice, embrace the challenges and opportunities that the above-mentioned workplace shifts have presented. For instance, they must engage in appropriate (e.g. brief, job-focused) counselling, always ensuring that they are attending to the employee’s psychological challenges and symptoms of distress and considering the possibility that they might need to refer out to a mental health specialist. Industrial-organisational psychologists must also evaluate and, if necessary, update their skills and abilities in relation to current employee and organisational needs.

### Limitations and recommendations

Three specific limitations must be borne in mind when evaluating the findings of this study. Firstly, our findings are based exclusively on the opinions and self-reported counselling skills of the IOPs; our data collection did not include direct observation of their workplace activities. Secondly, all references to postgraduate training of IOPs are based on the perceptions of the study’s participants; we did not review training curricula to ascertain the validity of those perceptions. Thirdly, our data and findings focus only on the preparedness and responsiveness components of emergency management. Future research could focus on how IOPs could ensure recovery and resilience for sustained employee mental health.

An alternative interpretation of the data we collected is that the contemporary role of the IOP does not involve counselling at the individual level; it is not viewed by them as an effective use of either their time or their skills. We recommend further research and investigation into this matter.

## Conclusion

The findings in this study highlight how COVID-19 has brought to the forefront the need for IOPs and organisations to place an emphasis on employees’ mental and physical health and well-being at all times. Although we found that IOPs generally responded to employee mental health needs in a positive manner, there was a lack of counselling preparedness and responsiveness during the COVID-19 pandemic. What seemed to be a key Rubicon was the participants’ conviction about counselling. This resulted in strong opinions on whether they will engage in (and grow skills in) the provision of counselling. The need to deal with employee mental health also posed particular challenges to participants in their personal capacity (psychological ill-preparedness), challenges that were compounded by a lack of existing organisational systems that could support the counselling process. We conclude that a concerted effort needs to be made to equip and empower IOPs as counsellors in the workplace so as to ensure preparedness for a rapidly changing environment.
